# Mandatory Disclosure of Pharmaceutical Industry-Funded Events for Health Professionals

**DOI:** 10.1371/journal.pmed.1000128

**Published:** 2009-11-03

**Authors:** Jane Robertson, Ray Moynihan, Emily Walkom, Lisa Bero, David Henry

**Affiliations:** 1 School of Medicine and Public Health, The University of Newcastle, Newcastle, New South Wales, Australia; 2 Department of Clinical Pharmacy, University of California San Francisco, San Francisco, California, United States of America; 3 Institute for Clinical Evaluative Sciences, Toronto, Canada

## Abstract

David Henry and colleagues examine compliance with new disclosure requirements of Medicines Australia, the pharmaceutical industry representative body, and argue that they fall short and instead more comprehensive reporting standards are needed.

**Summary Points**

- There are moves internationally to ensure greater disclosure of gifts and educational events for doctors paid for by pharmaceutical manufacturers. However, there is no agreement on appropriate standards of disclosure. In Australia, since mid-2007, there has been mandatory reporting of details of every industry-sponsored event, including the costs of any hospitality provided.
- Examination of the Australian data shows that although expenditure at individual events is often modest, cumulative expenditure is high, particularly in the case of medical specialists prescribing high cost drugs—oncologists, endocrinologists, and cardiologists.
- Although a significant advance, the new Australian reporting standards do not allow assessment of the educational value of sponsored events, and do not include details of speakers or educational content for most events. However, doctors in training are often present at these events.
- At present, the standards of disclosure are inadequate and should not be tied to an arbitrary monetary value of gifts or sponsorship. Reporting standards should require the names of the speakers presenting, whether sponsors played a role in suggestion or selection of speakers or the development of the content of presentations, and the nature of any direct or indirect financial ties between the speakers and the sponsors.

## Background

We are in a period of unprecedented scrutiny of the relationships between the pharmaceutical industry and doctors [1]–[4]. Legislators are now considering how they might become involved in the regulation of these practices. This is a telling comment on the perceived failure of the medical profession to regulate itself and of self-regulation by industry. But reliable and comprehensive data on the nature and extent of industry sponsorship are rare. Several states in the US have mandatory disclosure laws for physician payments, but these data have proved difficult to access and analyse [5]. The US Congress is considering new mechanisms for revealing industry–professional interactions (the so-called “Sunshine” Acts) [6],[7].

One of the first countries to move towards greater transparency was Australia. The pharmaceutical industry representative body, Medicines Australia, has a self-regulatory Code of Conduct that sets standards for the ethical marketing and promotion of prescription pharmaceutical products for its member companies. In addition to monitoring of promotional activities, a Code of Conduct Committee adjudicates on complaints regarding pharmaceutical company activities [8]. In 2007, the Australian Competition Tribunal placed disclosure requirements on Medicines Australia. It approved that body's Code of Conduct for industry–professional relationships on the condition that details of every sponsored event, including the costs of any hospitality, were posted on their website [9],[10]. Reporting commenced in July 2007 and data are updated six monthly [8].

In this Policy Forum we examine the Australian data and argue that although a definite advance, the Australian disclosure requirements fall short of what is required. We propose more comprehensive reporting standards, which should have application to other settings and jurisdictions.

## Australian Experience of Pharmaceutical Company Disclosures

In Australia, the emphasis in disclosure is on monitoring the level and type of sponsorship of educational events rather than documenting the dollar value of gifts and other payments to physicians. Since 2007 pharmaceutical companies have been required to report all functions (educational events) provided or sponsored for health professionals. They are required to disclose the following: the venue; the professional status of attendees; a description of the function and duration of the educational content of events; the nature of the hospitality; the total cost of hospitality; the numbers of attendees; and the total cost of the function [11].

The first report, covering the period July to December 2007, provided details of 14,649 events (Table 1) [12]. This total is equivalent to almost 600 events per week nationally, at a cost of around AUD$1 million/week (US$879,074.00). Put another way, the pharmaceutical industry spends, on average, around AUD$1,000 annually on each doctor through sponsorship of such events. The top five companies in terms of the numbers of sponsored events were Astra Zeneca, Pfizer, Sanofi Aventis, Janssen Cilag, and Eli Lilly (Table 1). The most generous of the active companies (those with >100 functions in 6 months) was Bristol Myers Squibb, with an average cost per head of AUD$95.26. In contrast, Alphapharm (a generics manufacturer) sponsored 441 events (mostly in professional rooms with a sandwich lunch) at an average cost per head of AUD$18.24 (Table 1).

**Table 1:** Summary statistics for events sponsored by the most active pharmaceutical companies.

Company	Events Reported (*n*)	Details of Company-Sponsored Functions^a^ (% of All Functions Sponsored by the Company)			
		Journal Club or Grand Rounds	Hospital or Professional Rooms	Restaurant, Hotel, or Function Centre	Average Cost/Head (AUD$) Spent on Hospitality
**AstraZeneca**	1,310	43.0	61.3	35.1	$40.37
**Pfizer**	1,266	38.9	52.5	41.4	$34.81
**Sanofi Aventis**	1,119	21.6	66.8	29.0	$48.12
**Jannsen Cilag**	1,080	28.6	64.2	32.4	$33.96
**Eli Lilly**	940	17.4	60.2	38.1	$47.38
**Novartis**	927	10.4	79.9	17.7	$56.22
**Roche**	776	18.3	78.0	18.9	$29.25
**GlaxoSmithKline**	738	18.6	57.6	37.0	$37.24
**Merck Sharp Dohme**	734	20.0	74.0	23.6	$26.81
**Servier**	608	8.6	57.7	39.8	$48.35
**Wyeth**	501	26.7	45.7	51.9	$56.33
**Alphapharm**	441	0.0	89.3	10.7	$18.24
**Merck Serono**	397	6.8	77.8	15.6	$18.78
**Novo Nordisk**	372	13.4	73.9	23.4	$22.65
**Amgen**	357	22.4	68.3	27.2	$43.55
**Boehringer Ingelheim**	340	0.0	0.3	99.1	$69.80
**Organon**	275	17.1	49.5	46.5	$42.58
**Abbott**	249	16.5	75.5	22.5	$31.18
**Mundipharma**	205	37.1	57.6	36.1	$32.76
**Schering Plough**	190	15.8	23.2	74.2	$65.24
**Nycomed**	165	14.5	15.2	77.6	$77.10
**Bayer**	158	3.8	34.8	59.5	$47.44
**Allergan**	155	0.0	29.0	58.7	$55.09
**BristolMyersSquibb**	151	0.0	15.2	76.8	$95.26

The educational event reports were downloaded as pdf files and converted into Excel spreadsheets; a coding scheme was devised by two authors (EW and JR). The codes were designed to differentiate the events based on: the duration; type of event; whether there were continuing professional development (CPD) or medical education (CME) points awarded; the venue; the professional status of attendees; the hospitality provided; and the cost of the hospitality. A number of companies specifically stated they were “not responsible” for the educational content of some events and we coded separately for these. The “not responsible” code included descriptors such as “topic set by hospital,” “third party organisation,” “external training company,” or “sponsorship only.” A series of primary analyses were conducted in Excel, providing descriptive statistics about the events sponsored by each company, and overall summary statistics. Ethics approval was not required to examine these publicly available data.

a An independent audit of the first posting of educational events was commissioned by Medicines Australia, with 951 events identified as requiring review. Further information was requested on 312 events with 52 referred to the Code of Conduct Committee. Twenty-four events were found to be in breach of the Code, this number reduced to 21 after appeals of the decision [23].

Hospitality (food, beverages, travel, accommodation) accounted for around AUD$17 million of the total of AUD$31 million spent on functions. Thirty-five percent of sponsored events (*n* = 5,174) were held in restaurants, hotels, or function centres. The average cost per head was much higher when the venue was a restaurant (AUD$71.35) than in a hospital (AUD$12.11). In 7.2% of cases (*n* = 1,062) expenditure exceeded AUD$100 per head (examples are given in Box 1). There were 74 events (0.5%) with total outlays per head on hospitality in excess of AUD$500.

**Box 1. Five examples of high-cost sponsored events**

1. Flights, accommodation, food, beverages, and conference registration fees for six ophthalmologists to attend a two-day conference in Spain, at a cost of AUD$10,993 per person, sponsored by Novartis.
2. One-hour cocktail party for 45 respiratory physicians on the Gold Coast, with hospitality costs of more than AUD$20,000, including flights and accommodation for one speaker, sponsored by Actelion.
3. A presentation by a Key Opinion Leader exploring the link between diabetes, severe mental illness, and antipsychotics for better patient management for 115 psychiatrists, general practitioners, and allied mental health workers at the RACV Cluba in Melbourne with a hospitality cost of AUD$186 a head sponsored by Eli Lilly. This amount included travel, accommodation, and extra meals for the speaker and 11 delegates.
4. Ten infectious diseases specialists given AUD$1,000 each, to contribute to flights, accommodation, and registration for a conference at Conrad Jupiter's Casino, Gold Coast, sponsored by Novartis.
5. Eight general practitioners attended an event with 2 hours of education at the Truffleduck restaurant in Perth, and earned 30 CPD points, with hospitality costs of almost AUD$140 a head, sponsored by Merck.
  Royal Automobile Club of Victoria.

Medical specialists were present at 62% (*n* = 9,018) of events, family physicians at 30% (*n* = 4,437), nurses at 26% (*n* = 3,820), and pharmacists at less than 5% (*n* = 621) of events. Registrars (medical specialists in training) were present at 19% (*n* = 2,827) of events; in 179 instances they were the only attendees. The medical subspecialties most often featured were psychiatry (17.9%), and oncology (15.2%), who received industry hospitality roughly three times as often as any other subspeciality (Table 2). The largest per head expenditure was directed at endocrinologists, oncologists, and cardiologists (Table 2). Companies spent considerably more on restaurant meals for doctors (AUD$76.73) than for nurses (AUD$48.78).

**Table 2:** Details of events where only specialists were present (*n* = 3,377 events).

Specialty	Number of Events	Percent	Average Cost/Head (AUD$) Spent on Hospitality
**Psychiatry**	606	17.9	$49.14
**Oncology**	514	15.2	$71.53
**Surgery**	221	6.5	$15.73
**Cardiology**	193	5.7	$70.50
**Anaesthesiology**	175	5.2	$26.58
**Neurology**	170	5.0	$63.11
**Endocrinology**	166	4.9	$71.77
**Haematology**	156	4.6	$41.76
**Pathology**	148	4.4	$14.16
**Radiology**	138	4.1	$12.44

Companies reported no responsibility for the educational content in only 9% of events (*n* = 1,287). Likewise, continuing medical education (CME)/continuing professional development (CPD) points were allocated to 9% of events (*n* = 1,270). Just over 20% of all events were described as “journal club” or “grand rounds” (*n* = 3,035), mostly conducted in hospitals. The majority of events (*n* = 10,723, 73.2%) were a mix of meetings of various kinds, including workshops and in-service training activities; only 4% (*n* = 591) were described as “conferences.” Table 3 shows the topics discussed, the most common being cardiology, diabetes, oncology, psychiatry, and respiratory medicine. The most common specific topics were hypertension, osteoporosis, breast cancer, type-2 diabetes, and depression. All represent large and important markets for pharmaceutical products. Topic descriptions, where provided, often matched the product portfolio of the sponsor, although there were few mentions of specific drug names (*n* = 582, 4%).

**Table 3:** Ten most commonly reported topic areas covered in company-sponsored events.

Area^a^	*n*
Cardiology	1,085
Hypertension	266
Lipid lowering	112
Pulmonary arterial hypertension	69
Diabetes	1,075
Type 2 diabetes	192
Insulin and devices	107
Type 2 diabetes: blood pressure control	55
Oncology	1,041
Breast cancer	193
HPV/cervical cancer	101
Colorectal cancer	68
Prostate cancer	59
Psychiatry	967
Depression	170
Psychosis	99
Bipolar disorder	84
Respiratory	588
Asthma	143
Chronic Obstructive Pulmonary Disease	71

a Each event could cover more than one topic.

HPV, human papillomavirus.

Importantly, Australian companies are not required to disclose the names of the speakers, whether sponsors played a role in their selection or in the choice of the content of presentations. They are also not required to disclose the nature of any financial ties between their companies and the speakers.

## Why Do We Need Better Disclosure?

The information provided by Medicines Australia points to a high level of contact between pharmaceutical manufacturers and health professionals, particularly doctors. The per-person expenditure was greatest for medical specialists who prescribe high cost drugs—oncologists, endocrinologists, and cardiologists. Generally, expenditure at individual events was modest; however the cumulative expenditure and the overall level of contact was high. The available information suggests that companies exert influence over the educational content of events in most cases, and doctors in training are often present at these functions. There is substantial evidence that attendance at company-sponsored events modifies prescribing practices [13]–[15]. The presence of doctors in training and students (in hospital-based sessions) may lead to a process of enculturation whereby they come to regard repeated contact with pharmaceutical companies as a normal and acceptable part of their professional practice. The data reviewed here indicate that, from a company perspective, it is cheap and easy to sponsor meetings in hospitals and health centres, and the return on this “investment” is likely to be high. Equally, it is straightforward for administrators to limit sponsorship of such activities, should they choose to do so. It is difficult to see a role for pharmaceutical companies at hospital grand rounds.

The evidence from this analysis of Australian data suggests that disclosure requirements should not stipulate thresholds—set dollar amounts below which disclosure is not required. Physician-reporting requirements such as those in Vermont and Minnesota in the US, which exempt payments of less than US$100, could obscure the broad cumulative influence of a number of smaller payments [5],[16]. The literature indicates that it is not only the size of the gift that matters—it is the sense of reciprocity that it engenders [17].

The types of activities described here need to be viewed within the broader context of other forms of pharmaceutical industry interaction with doctors, including face-to-face contact with representatives, advertising in medical journals, consultancies, membership of advisory boards, and stock holding [18]–[20]. While lavish gifts and generous travel support have been a focus of attention in the past, these have been progressively discouraged by industry and professional guidelines. It is likely that the frequent, more modest, sponsored educational events will become increasingly important and influential, and the principal form of contact between industry and health professionals.

There are a number of organisations that will benefit from more comprehensive disclosure of these activities. Professional organisations and accreditation bodies will have accurate data on the level and type of contact their members have with pharmaceutical companies. This will enable them to counter the undesirable effects of such relationships through the development of guidelines, or the evolution of practice standards or disciplinary codes. They will benefit from sequential data to determine if practices are changing over time. The public, the media, and consumer groups will have access to reliable data on which to base their judgements about industry-health professional contact and, when appropriate, to lobby for change. Individual health professionals could have access to information on which to judge their own practices against those of their peers. If legislation is thought necessary, governments will have data on which to monitor its impact.

## Proposals for Greater Transparency

The Australian reporting standards are deficient in not including details that enable a judgement about the educational value of company sponsored events. We believe that reporting schemes should require the following details: the names of the speakers presenting, whether sponsors played a role in suggestion or selection of speakers or the development of the content of presentations, and the nature of any direct or indirect financial ties between the speakers and the sponsors. This type of information is routinely requested by professional journals; so there are ample precedents and it is particularly relevant when judging the appropriateness of educational events.

We experienced considerable difficulty in accessing the Australian data, which are compiled in portable document format (pdf). As suggested in the US Sunshine Acts it is important that summary reports listing each function are accessible to the public in a searchable, downloadable, and analysable format [5]–[7].

Whether there should be a central register or database that identifies attendees at company-sponsored functions is more controversial. The data could be compiled from the records of names collected by the pharmaceutical companies. Reports could be provided to health professionals, which would enable them to compare their practices with their peers. We are not here advocating public disclosure of this information, but individuals could be asked to provide reports in particular circumstances—for instance when ethics committees are considering the industry ties of an investigator.

In Box 2 we have summarised the main data elements that we think should be included in disclosure programs. What we suggest is consistent with the recent Institute of Medicine (IOM) Report on conflicts of interest [21]. This report recommended that the US Congress create a national program requiring companies and their foundations to publicly report payments to physicians and other prescribers, biomedical researchers and their institutions, but did not suggest specific data elements. Some authors of the report argued that this database should also provide explanatory material about payments received (e.g., for an educational or marketing purpose) and information on all financial ties (e.g., equity ownership, patent rights) in addition to industry payments and gifts [22].

**Box 2. Details to be included in mandatory reporting schemes for pharmaceutical industry–sponsored events for health professionals**

**Included in existing reports from Medicines Australia**

The numbers of attendees and their professional status

The venue, and a description of the function

The nature of any hospitality provided

The total cost of hospitality and the total cost of the function

The nature of any entertainment provided

The duration of the educational content of events

Continuing professional development (CPD)/continuing medical education (CME) points provided

**Suggested additional compulsory reporting items**

The nature of any gifts provided

The names of speakers

Dollar value of honoraria and travel support provided to speakers

Disclosure of other financial ties between sponsoring companies and speakers (e.g., equity ownership, consultancies, advisory panel membership)

The role of the company in suggesting/choosing the educational topic and speaker

The brand names of drugs discussed in the session

**For debate**

Registration of all attendees (limited access [Information available only to the individual and through him or her to other bodies])

While it may be unrealistic and undesirable to ban contact between pharmaceutical companies and health professionals we should work to make those relationships completely transparent. We welcome further debate on this topic.
